# Perspective: Vitamin D deficiency and COVID‐19 severity – plausibly linked by latitude, ethnicity, impacts on cytokines, ACE2 and thrombosis

**DOI:** 10.1111/joim.13149

**Published:** 2020-07-22

**Authors:** J. M. Rhodes, S. Subramanian, E. Laird, G. Griffin, R. A. Kenny

**Affiliations:** ^1^ From the Department of Cellular and Molecular Physiology Institute of Translational Medicine University of Liverpool Liverpool UK; ^2^ The Irish Longitudinal Study on Ageing School of Medicine Trinity College Dublin Dublin Ireland; ^3^ Infectious Diseases and Medicine St George’s University of London London UK; ^4^ Department of Medical Gerontology Mercers Institute for Ageing St James Hospital Dublin 8 Ireland

**Keywords:** vitamin D, COVID‐19, cytokine

## Abstract

**Background:**

SARS‐CoV‐2 coronavirus infection ranges from asymptomatic through to fatal COVID‐19 characterized by a ‘cytokine storm’ and lung failure. Vitamin D deficiency has been postulated as a determinant of severity.

**Objectives:**

To review the evidence relevant to vitamin D and COVID‐19.

**Methods:**

Narrative review.

**Results:**

Regression modelling shows that more northerly countries in the Northern Hemisphere are currently (May 2020) showing relatively high COVID‐19 mortality, with an estimated 4.4% increase in mortality for each 1 degree latitude north of 28 degrees North (*P* = 0.031) after adjustment for age of population. This supports a role for ultraviolet B acting via vitamin D synthesis. Factors associated with worse COVID‐19 prognosis include old age, ethnicity, male sex, obesity, diabetes and hypertension and these also associate with deficiency of vitamin D or its response. Vitamin D deficiency is also linked to severity of childhood respiratory illness. Experimentally, vitamin D increases the ratio of angiotensin‐converting enzyme 2 (ACE2) to ACE, thus increasing angiotensin II hydrolysis and reducing subsequent inflammatory cytokine response to pathogens and lung injury.

**Conclusions:**

Substantial evidence supports a link between vitamin D deficiency and COVID‐19 severity but it is all indirect. Community‐based placebo‐controlled trials of vitamin D supplementation may be difficult. Further evidence could come from study of COVID‐19 outcomes in large cohorts with information on prescribing data for vitamin D supplementation or assay of serum unbound 25(OH) vitamin D levels. Meanwhile, vitamin D supplementation should be strongly advised for people likely to be deficient.

## Graphical abstract

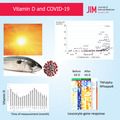

## Introduction

The SARS‐CoV‐2 coronavirus is an enveloped RNA virus, infection by which provokes a remarkable range of responses from complete lack of symptoms through to cytokine storm and life‐threatening acute respiratory distress syndrome (ARDS) [1, 2]. The explanations for this extremely variable prognosis are unclear. Mortality from coronavirus infectious disease 2019 (COVID‐19) is higher amongst people who are older, male, obese, diabetic, hypertensive, or who are from black, Asian, or minority ethnic (BAME) demographics. All these factors are associated with increased prevalence of vitamin D deficiency or, as in male sex, with reduced impact of vitamin D on the immune response. Vitamin D is, like cortisone and sex hormones, a cholesterol‐derived steroid hormone, and it modulates expression of around 5% of human genes including many relevant to the immune response to pathogens. We have therefore examined the evidence that vitamin D deficiency might be a factor determining severity of COVID‐19.

## Association between northerly latitude and increased mortality from COVID‐19

**Figure 1:**
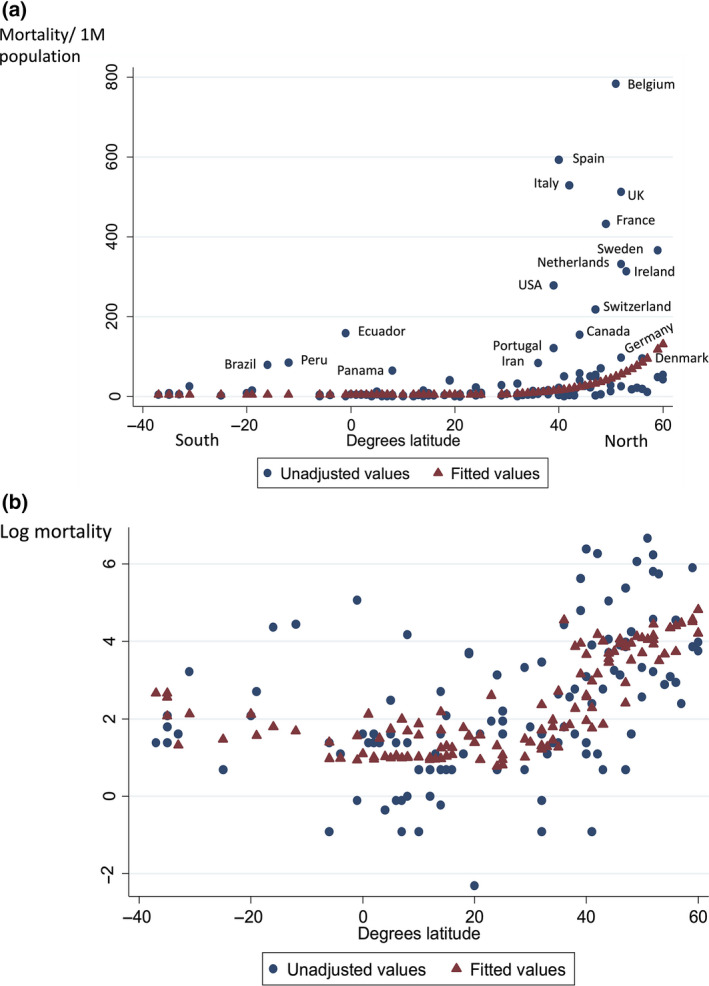
(a) COVID‐19 mortality per 1 million population by country compared with latitude of capital cities. Fitted values are derived from a piecewise linear model of the logarithm of mortality on latitude. This was based on a threshold of 28 degrees North that explained the greatest amount of variation. (b) Logarithm of COVID‐19 mortality per 1 million compared with latitude with and without adjustment for age (%≥65 years). Data accessed 18 May 2020. Reproduced from [3] with permission.

There is currently (May 2020) a significant association between northerly latitude and mortality from COVID‐19 expressed per million population across the 117 countries with more than 1 million population and more than 150 recorded cases at time of sampling (Fig. 1) [3]. Much of this association is due to the younger age of populations in some countries. Adjusting for per cent of population ≥ 65 years does however leave a significant relationship between latitude and COVID‐19 mortality (*P* = 0.031) with an estimated 4.4% increase in mortality for each 1 degree latitude north of 28 degrees North (Table 1). Addition of neither pollution (particles of matter < 2.5 µm diameter (PM_2.5_) micrograms per m^3^) nor population density per country added significant explanatory power to a model containing latitude and age. An association between northerly latitude and mortality has also been noted amongst African Americans across the United States [4].

**Table 1:** Associations between COVID‐19 mortality by country, latitude and % of population ≥ 65 years (from [3], data accessed 18 May 2020).

Variable	Regression coefficient	Standard error	*P*‐value	% of variation explained	Effect size (95% CI)^a^
*Univariate models*					
Latitude	0.1074	0.0142	<0.0005	33.1	11.3% (8.3–14.5%)
% ≥65	0.1766	0.0199	<0.0005	40.4	19.3% (14.8–24.1%)
*Multivariate model*					
Latitude	0.0428	0.0196	0.031	43.0	4.4% (0.4–8.5%)
% ≥65	0.1281	0.0291	<0.0005		13.7% (7.4–20.3%)

^a^ The effect size is, for latitude, the percentage increase in mortality from one location, situated at least 28°north, to another location one degree further north and, for % ≥ 65, the percentage increase in mortality for each one % increase in % ≥65

Associations between COVID‐19 mortality and latitude suggest a possible effect of ultraviolet light. A substantial source of vitamin D comes from synthesis in the skin from its precursor 7‐dehydrocholesterol as a consequence of ultraviolet light (UVB) exposure. People living far from the equator may therefore become vitamin D deficient in the winter and spring, with levels lowest from December to May [5]. It is estimated that at latitudes below 35 degrees, either side of the equator UVB radiation is sufficient for year‐round vitamin D synthesis, although this will also depend on diet, skin colour, clothing, time spent outdoors and use of sunscreen [6].

Interpretation of country‐to‐country variation is further complicated by variable approaches to supplementation and vitamin D food fortification initiatives. Thus, vitamin D levels are generally well maintained despite relative lack of UVB exposure in Nordic countries due to widespread use of supplements and food fortification, whereas deficiency is commoner in the United Kingdom and in southern European countries, [7, 8] and particularly amongst persons over 80 years and those in institutions. We have recently reported a significant correlation between COVID‐19 mortality and reported average serum vitamin D levels across European countries [9]. Vitamin D deficiency defined as <30 nmol L^−1^ is found in over 10% of Europeans [7], but it has been suggested that a level of at least 50 nmol L^−1^ may be optimal [6]. Wuhan itself, where the outbreak started, is at latitude 31 degrees north; however, air pollution is also a major factor limiting UVB radiation and has previously been very marked over this densely populated (11M) city [10]. There are currently no population‐based vitamin D data available from Wuhan. In sunnier Brazil, whose capital Brasilia is at −16 degrees latitude, there is now high mortality but meta‐analysis has shown 28% prevalence of vitamin D deficiency [11].

Alternative explanations for the north‐south gradient in COVID‐19 mortality are arguably less plausible. Although population density expressed per country does not currently associate with COVID‐19 mortality, it could be speculated that cities tend to be smaller and urban populations less densely crowded further south but there are many examples of high population cities below 28 degrees latitude north – Karachi 14.9M, Hong Kong 7.4M, Mexico City 8.9M, Nairobi 4.3M and Sidney 5.2M, for example. It can also be pointed out that there is a north‐south gradient for diagnosed cases, however, if true – for diagnosis rate is of course very dependent on testing frequency ‐ this may well reflect the longer period of infectivity that is associated with more severe COVID‐19 illness. It does not seem very likely that the virus has simply had less opportunity to spread south of the equator given that we are now 4 months into the pandemic.

Ultraviolet light, particularly UVB, has also been shown to have direct immunosuppressive effects on the skin, that include suppression of contact sensitivity at the UV‐irradiated site and induction of antigen‐specific tolerance mediated by regulatory T lymphocytes. Significant systemic immunosuppression has also been demonstrated in experimental models [12]. Various mediators are thought to be responsible for these effects. These include *cis*‐urocanic acid, generated by the isomerization of *trans*‐urocanic acid, which binds to the serotonin receptor on antigen‐presenting cells, keratinocytes and mast cells, and also cyclobutene pyrimidine dimers, generated by UV‐mediated nucleotide damage, and oxidation products of membrane lipids.

UV light could also reduce viability of free virus in the environment. Although UVC light (200–280 nm wavelength) that has a strong germicidal effect does not penetrate the earth’s atmosphere, UVB (280–320 nm) has a weaker but significant anti‐viral effect that may shorten the survival of the virus on surfaces and thus reduce infection rates [13]. Higher temperature and higher humidity can also decrease viral survival in the environment and have been shown to correlate with reduced COVID‐19 infection rates and mortality across 166 countries [14]. The evidence is contradictory though: a recent study in the United States has linked UVB, and higher temperature, but not rainfall with lower SARS‐CoV‐2 infection rates [15], whereas a study across Chinese cities has shown no association with either UVB or temperature and *R*_0_ or infection rates [16].

Thus, although the association with latitude implies that COVID‐19 may prove seasonal, the mechanisms underlying this could include any or all of impacts of UVB on the immune system mediated by vitamin D synthesis, other consequences of the actions of UVB in the skin and direct effects on environmental survival of SARS‐CoV‐2 consequent to UVB, humidity or temperature. Of these, only an effect of UVB mediated by vitamin D synthesis would readily explain the associations between COVID‐19 mortality and ethnicity.

## Vitamin D chemistry and biology

**Figure 2:**
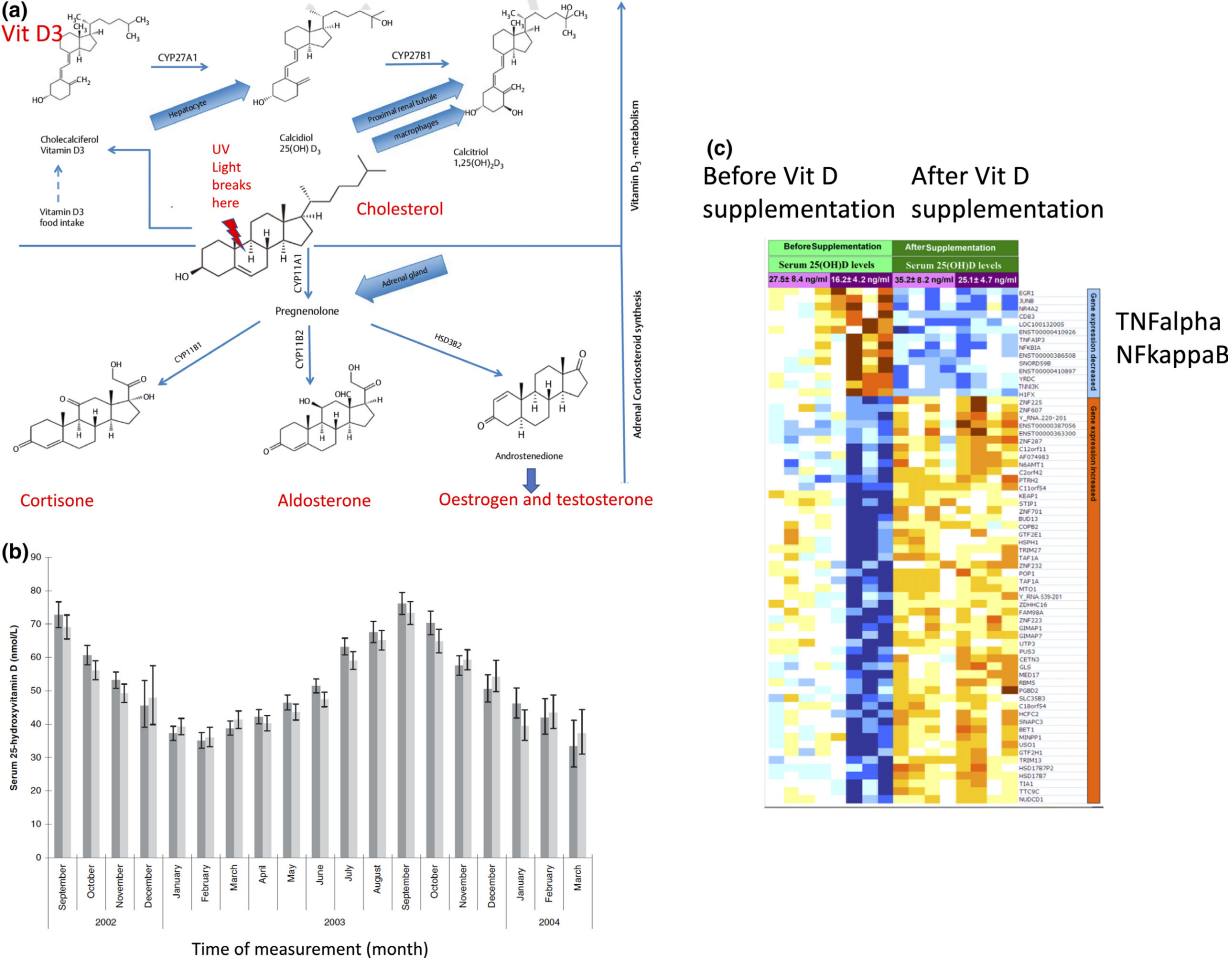
(a) Synthesis of vitamin D, adrenal and sex hormones from cholesterol. Adapted from Muscogiuri et al. [17] with permission. (b) Seasonal variation in serum vitamin D levels (mean [95% CI]) amongst 7437 white British (1958 British birth cohort) at age 45. Dark bar = male, pale bar = female. From Hypponen and Power [22] with permission. (c) Heatmaps of vitamin D‐responsive genes in mixed leucocytes from individuals before and after 2 months of vitamin D supplementation. Four individuals had prior vitamin D deficiency, and 4 individuals had prior normal levels. Blue = decreased expression. Brown = increased expression. It can be seen that vitamin D responsiveness equalized between the two sets of individuals after supplementation. From Hossain‐nezhad et al. [28] with permission.

Vitamin D, uniquely amongst the vitamins, is a steroid hormone. It is fat‐soluble and exists in two forms, vitamin D2 (ergocalciferol) and vitamin D3 (cholecalciferol). Both are generated by the action of UVB, splitting a single (9,10) carbon–carbon bond in their respective precursors – ergosterol and cholesterol thus generating a secosteroid or ‘cut’ steroid. Ergosterol is the precursor in fungi and plankton and cholesterol the precursor in animals. The chemical structure of vitamin D therefore has close similarities with that of the other cholesterol‐derived hormones such as cortisol, aldosterone, testosterone and oestrogen (Fig. 2). Vitamin D3 has greater affinity for the circulating vitamin D binding protein and a substantially longer half‐life in the circulation than vitamin D2 [18]. This is probably only of major significance if taken by intermittent bolus dosing rather than daily supplementation [19]. After the generation of cholecalciferol (or ergocalciferol), further hydroxylation is required in the liver and then the kidney to generate the active 1,25 dihydroxycholecalciferol. It should be noted though that macrophages/dendritic cells also have the ability to convert 25(OH)D to 1,25(OH)_2_D via CYP27B1 and that clinical consequences of vitamin D deficiency correlate better with serum concentration of 25(OH)D rather than with the (1000‐fold lower) serum concentration of 1,25(OH)_2_D [20]. Although lymphocytes also express CYP27B1, this is at substantially lower level and the regulatory (anti‐inflammatory) effect of vitamin D on human lymphocytes in mixed cell culture requires the presence of antigen‐presenting dendritic cells [21]. The daily requirement of vitamin D is estimated at between 5 and 20 µg (200 to 800 IU) [6], and it is not easy to achieve this through diet alone. Oily fish is the only really substantial natural dietary source though farmed versus wild fish concentrations vary. Liver and eggs also contain vitamin D but a single egg only provides about 5% of the daily requirement. Mushrooms need to be subjected to UV irradiation and even then will only provide modest amounts of vitamin D2. For the majority of people, the main source of vitamin D is its generation by the action of UVB on cholesterol in the skin. This is evidenced by the fact that in the UK, blood levels of vitamin D are approximately 50% lower in February than in September [22] (Fig. 2). That seasonal differences are not even greater reflects the long half‐life of vitamin D in the body (2–3 months), predominantly in fat stores, in contrast to its relatively short half‐life in the blood (2–3 weeks) [23].

Vitamin D is best known for its effects on calcium and phosphate absorption, osteoclast activation, and hence on bone calcification and muscle strength [24]. However, the vitamin D receptor is very widely expressed, including by all leucocyte classes [25].

In the blood, approximately 85% of vitamin D is bound to vitamin D binding protein (DBP), 15% to albumin and just 0.03% of 25(OH)D_3_ and 0.4% of total 1,25(OH)_2_D_3_ are free vitamin D [26]. It is thought that in most cells, only free vitamin D can enter the cell. Cellular entry by protein‐bound vitamin D is dependent on expression of the cell surface receptor proteins megalin and cubilin [20] and is largely restricted to the kidney, parathyroid and placenta. Free vitamin D diffuses through the plasma membrane and binds to the vitamin D receptor (VDR) in the cell nucleus where the vitamin D/VDR complex then interacts with vitamin D response elements in the genome. It is estimated that vitamin D affects the transcription of around 1000 genes, that is around 5% of the human genome [27]. In pooled leucocytes, it has been shown that at least 60 genes are vitamin D‐responsive [28] and nearly two hundred genes in monocyte/macrophage cells [29, 30, 31] (Fig. 2). Vitamin D therefore has substantial effects on the immune system that are highly relevant to the response to pathogens.

## Impact of vitamin D on immunological response to pathogens – clinical studies

A protective effect of cod‐liver oil in tuberculosis was recognized in an early therapeutic trial conducted at London’s Brompton hospital in the 1840s [32] and the role of vitamin D in this effect was recognized in the 1940s, initially with the successful treatment of cutaneous tuberculosis and subsequently with many confirmatory studies [33]. Since then, knowledge of the impacts of vitamin D on the immune system has expanded greatly and there has been increasing recent focus into its role in determining response to viral infections, particularly respiratory viruses.

The evidence both from laboratory studies and from clinical studies is that vitamin D status has probably only a small impact on risk for viral infection but a much more important impact on inflammatory response and hence severity. Thus, a meta‐analysis looking at impact of supplementary vitamin D on risk for upper respiratory tract infection showed a statistically significant but very modest reduction, from 42.2% to 40.3%, in risk of one or more infections [34]. However, amongst those who were vitamin D deficient at baseline the reduction in infection rate was greater – from 55.0% down to 40.5%. A beneficial effect was only seen with regular daily dosing and not with intermittent bolus dosing. Probably more impressive is the association between vitamin D deficiency and severity of respiratory disease – for example the need for intensive care in 1016 infants hospitalized with bronchiolitis (22% if vitamin D < 20 ng mL^−1^ (50 nmol L^−1^), compared with 12% if vitamin D > 30 ng mL^−1^ (75 nmol L^−1^); *P* = 0.003) [35].

A study in Irish people >60 years old and healthy apart from hypertension showed a strong correlation between vitamin D deficiency and increase in both IL‐6 and C‐reactive protein [36]. A detailed investigation of the impacts of vitamin D, gender and seasonality on cytokines has subsequently been undertaken in 534 healthy subjects as part of the Human Functional Genomics Project [37]. This showed that monocyte inflammatory cytokine responses to lipopolysaccharide and *Candida albicans* are substantially greater in men. Several inflammatory cytokines, including TNF‐alpha, interleukin beta and interleukin 6, were shown to be higher in summer, mostly showing no relationship with vitamin D.

## Interpreting serum vitamin D levels during illness – the negative acute phase effect

Studies of vitamin D levels in individuals who are already ill or have raised inflammatory markers have to be interpreted with caution. Controlled studies in calves infected with bovine diarrhoea virus (BVDV) showed that serum vitamin D levels fell by 57% during the acute phase response to illness [38] and similar falls have been documented in humans following orthopaedic surgery and acute pancreatitis [39]. Both serum vitamin D binding protein (DBP) and albumin concentrations fall in illness and total vitamin D levels will fall as a consequence. It is therefore almost inevitable that there will be a correlation between lower total serum vitamin D levels and increased COVID‐19 severity. There are several possible ways of getting around this problem:

### Measurement of free vitamin D

Total 25(OH)D serum concentration in serum is generally acknowledged to be the *de facto* biomarker of vitamin D status. However, as already noted, the unbound (i.e. free) concentration of 25(OH)D is below 0.5% of the total concentration [26, 40]. Emerging evidence points to a critical role for free, rather than total 25(OH)D in mediating important cellular processes related to immunity. For instance, *in vitro* studies have demonstrated reduced immune functions of dendritic cells [21] and adherent monocytes [41] by increasing DBP in culture media which reduces the amount of free 25 (OH)D. This is analogous to free thyroid hormones or testosterone which are physiologically more relevant than their total concentration.

Measurement of free 25(OH)D has been challenging due to its very low serum concentrations (approximately 10‐fold less than free thyroid hormones) and has historically relied on cumbersome radioactive tracer‐based methods [42]. More recently, two further assays, including an ELISA [43] and a high‐throughput mass spectrometric method [44] for direct measurement of free 25(OH)D, have been introduced, but require further clinical and technical validation. Thus, computational methods which rely on concentrations of total ligands and DBP and their *in vitro* measured affinity constant are often used to calculate free 25(OH)D [45]. It is important to note, however, that the biological significance of DBP’s various allelic forms on DBP concentrations and affinity differences is yet to be fully established. The experimentally measured affinity differences for vitamin D metabolites for various genotypic forms of DBP with the exception of one study [46] appear to be small [47, 48]. On the other hand, genotype has been consistently shown to alter serum DBP concentrations [49, 50]. In summary, the main role of DBP in determining free 25(OH)D levels appears to be DBP concentration‐dependent rather than genotype‐dependent. Thus, measurement of serum DBP and albumin alongside total 25(OH)D should allow a robust computational approach for calculating free 25(OH)D and permit a better correlation between vitamin D status and COVID‐19 severity.

### Associations with vitamin D receptor polymorphism

Vitamin D receptor polymorphisms impact on vitamin D response. Meta‐analysis has shown a highly significant relationship (*P* = 0.007 OR 1.52) between hospitalization for respiratory syncytial virus (RSV) bronchiolitis and possession of a minor allele for a vitamin D receptor polymorphism (Fok1‐f rs2228570) that lowers transcriptional activity of the vitamin D receptor [51]. This polymorphism has an allele frequency of 13 to 38% in healthy subjects so it could be very informative to know whether this allele is also seen with higher frequency in people with more severe COVID‐19.

### Mendelian randomization

The difficulty in interpreting serum vitamin D levels during illness has led investigators to consider the application of Mendelian randomization. This uses gene polymorphisms that predict vitamin D status as a surrogate for vitamin D deficiency. One approach that has proved successful has used gene polymorphisms associated with risk of skin colour, tanning, or freckling. This identified a group of gene polymorphisms that together were predictive of vitamin D status [52]. However, this included genes such as HERC2 that are major determinants of blue eye colour [53] which in turn are strongly associated with reduced pigmentation and enhanced vitamin D response to UVB in white individuals [54]. This approach may therefore be less effective in a population that contains mixed ethnicities.

A broader statistical approach is to use a genome‐wide association study (GWAS) to identify, in a hypothesis‐free fashion, polymorphisms that associate with vitamin D deficiency. This has been done in a remarkable study across 79,366 European‐ancestry individuals [55]. Polymorphisms at six loci were informative with high significance. However, the overall estimate of heritability of serum vitamin D concentrations was found to be only 7.5% and with only 38% of that heritability accounted for by the identified polymorphisms. This approach will therefore only be useable with very large sample sizes. Moreover, a different GWAS would need to be performed for other ethnic groups.

### Measurement of vitamin D in hair or other tissues

Study of vitamin D levels in hair samples has been proposed as a way of avoiding the negative acute phase response effect of severe illness on serum vitamin D levels [56]. This approach is proving reliable in measurement of other steroid hormones such as cortisol [57, 58] but would need further validation.

## Impact of vitamin D on immunological response to pathogens – laboratory studies

In keeping with the clinical studies, experimental evidence shows that vitamin D has shown only inconsistent effects on viral replication in human respiratory epithelial cell culture but markedly down‐regulates production of pro‐inflammatory cytokines including TNF‐alpha and IL‐6 by various mechanisms including inhibition of viral‐induced NF‐kappaB activation [59].

Vitamin D receptors are expressed by most immune cells including activated T cells, B‐cells and dendritic cells, and macrophages. Vitamin D is important for killing of phagocytosed bacteria, including *Mycobacterium tuberculosis* [60, 61] and *E. coli*, [62] by macrophages. An important part of this bactericidal effect relates to the induction by vitamin D of cathelicidin, a cationic bactericidal peptide [63]. Cathelicidin (LL‐37) can be produced not only by macrophages but also by epithelial cells and has been shown to have anti‐viral activity, particularly against enveloped viruses [64]. Vitamin D has been shown to induce an anti‐viral effect against rhinovirus in cultured respiratory epithelial cells [65], an effect that can also be demonstrated by addition of exogenous cathelicidin [66]. An effect of cathelicidin against influenza has also been shown [67]. Currently though, the impacts of vitamin D on macrophage defence against viral pathogens have demonstrated a predominant impact on cytokine response rather than on viral killing [68]. Some of the work has focussed on Dengue fever, a viral infection that is well known for its very marked cytokine activation and risk of organ failure [69, 70], although vitamin D deficiency has paradoxically been shown to correlate with reduced risk of septic shock in Dengue fever [71]. Experiments have shown consistent suppression of inflammatory cytokine response to pathogens by vitamin D, in macrophages, and also in T cells and in various animal models of pneumonia and pneumonitis [72, 73, 74]. Cytokines suppressed include IL‐6 that has been incriminated in COVID‐19‐associated ARDS.

Given that vitamin D may regulate the response of nearly two hundred genes in the monocyte/macrophage, it is not surprising that its suppressive effect on macrophage cytokine responsiveness has been shown to be effected via more than one pathway. Vitamin D has been shown to regulate the production of inflammatory signalling mediated by both NF‐kappaB and STAT‐1 [75] with MAPkinase activation as an important precursor [72]. There has therefore been increasing speculation that vitamin D deficiency could contribute to a risk of more serious COVID‐19 disease with increased risk of cytokine storm and consequent acute respiratory distress syndrome (ARDS) [76, 77].

## Vitamin D immune response and gender

The impact of vitamin D on suppressing the immune response has been shown to differ between men and women. Vitamin D induces reduction in pro‐inflammatory cytokines IL17 and interferon gamma and increase of interleukin 10 production by CD4 + T lymphocytes, effects that are much greater in T lymphocytes from women than from men. Similarly, anti‐CD3‐ and anti‐CD28‐stimulated peripheral blood mononuclear cells from men generated less than half the number of regulatory CD4 + CD25 + FoxP3 + T lymphocytes in response to vitamin D compared with cells from women, but this gender difference disappeared when oestradiol was added [78]. In keeping with this, a Norwegian study has looked at the effect of weekly supplementation with 20 000 IU vitamin D3 or placebo on the human transcriptome in prediabetic individuals with impaired glucose tolerance. Fifty‐eight genes were shown to be significantly affected by vitamin D in men compared with 185 in women (*P* < 0.05). In women, 51 genes showed a 2‐log difference in expression compared with only a single gene in men [79]. Genes affected included those related to the interleukin signalling pathway and B cell‐mediated immunity. The authors speculated that the gender difference might be related to oestrogen‐dependent effects on synthesis of the vitamin D binding protein.

## Increased prevalence of vitamin D deficiency amongst people with risk factors for severe COVID‐19 including ethnicity, diabetes, hypertension, obesity and institutionalization

Vitamin D deficiency is commoner in obese individuals, people with type 2 diabetes, hypertension, and most strikingly amongst ethnic minorities in Europe and North America – where darker skin pigmentation reduces skin synthesis and eightfold increased prevalence of deficiency is reported (Table 2) [80, 81, 82, 83]. All of these are demographics that have been associated with increased risk of severe COVID‐19. Vitamin D deficiency is also substantially commoner amongst people who are institutionalized including prisoners and people in care homes [84, 85, 86, 87]. Since vitamin D is fat‐soluble, its deficiency is also more likely in people with chronic digestive disorders such as Crohn’s disease or chronic pancreatitis but, hopefully, most will be receiving supplements.

**Table 2:** Associations between vitamin D status and demographic variables associated with COVID‐19 mortality

Author/year	Demographic variable	Type of study/location	*n*	Findings	Conclusions
Kunutsor et al 2013 ^80^	Hypertension	Meta‐analysis	283 537	Relative risk for hypertension reduced by 0.88 (95% CI 0.81–0.97) per 10 ng mL^−1^ increment in vitamin D levels	Inverse correlation between vitamin D status and hypertension
Mauss et al 2015 ^81^	Diabetes	Cross‐sectional (Germany)	1821	Vit D < 10 ng mL^−1^ associated with increasing HbA1c *P ≤ *0.001 And type 2 diabetes OR 2.55 (95% CI 1.16–5.12)	Strong inverse correlation between vitamin D status, fasting glucose, HbA1c and type 2 diabetes
Yao et al 2015 ^82^	Obesity	Meta‐analysis	13 209	Vitamin D deficiency (varying definitions) OR 3.43 (95% CI 2.33 to −5.06) for obesity	Strong inverse correlation between vitamin D status and obesity
Herrick et al 2019 ^83^	Ethnicity	Cohort study (USA)	16 180	Prevalence of vitamin D deficiency (<30 nmol L^−1^) 17.5% (95% CI 15.2–20.0) in non‐Hispanic black; 2.1% (95% CI 1.5–2.7) in non‐Hispanic white	Strong association between ethnicity and vitamin D deficiency. No gender difference

## Vitamin D, the renin–angiotensin system and COVID‐19

The receptor for SARS‐CoV‐2, as for SARS‐CoV, is angiotensin‐converting enzyme 2 (ACE2) so there is intense interest in factors that alter its expression or function. ACE2 has potentially contradictory roles. Given that it is the receptor for SARS‐CoV‐2, it would be reasonable to assume that greater expression of ACE2 would be bad for the human host. However, since the discovery of ACE2 twenty years ago various studies have shown that it has a crucial role in protecting against acute lung injury and ARDS in experimental models [88, 89, 90]. The balance between ACE2 and ACE seems crucial as ACE2 counteracts the effects of ACE by hydrolysing angiotensin II to angiotensin (1‐7). Since angiotensin II is central to the development of ARDS, this is a very important protective mechanism. So, more ACE2 is good – at least in respect of reducing risk of ARDS, and ACE2 also has a protective role against cardiovascular diseases [91, 92]. ACE2 is highly expressed on human lung alveolar cells but also on vascular endothelial cells, smooth muscle cells, renal tubular epithelium and small intestinal enterocytes. There is frustratingly little published information on its expression, or perhaps more importantly on the ratio of ACE2:ACE expression, in children, males, elderly, varying ethnicity etc other than very small human studies [93] or animal studies. The gene encoding ACE2 is carried on the X chromosome. Serum assays have shown no sex differences in ACE2 concentration overall but higher serum ACE2 in older women [94]. Studies in rats have however shown substantially reduced ACE2 expression with ageing and particularly in older males [95].

Vitamin D has been shown experimentally to increase ACE2, reduce ACE expression, reduce angiotensin II production and reduce damage in lipopolysaccharide (lps)‐induced lung injury in rats [96]. Similarly, vitamin D receptor gene knockout mice show much more severe acute lung injury and increased mortality in an lps‐sepsis model of ARDS with amelioration by antagonists of angiotensin II [73]. Vitamin D also suppresses expression of renin, the rate‐limiting enzyme in the renin‐angiotensin cascade [97]. These effects are clearly highly relevant to a potential role of vitamin D in protecting against ARDS in COVID‐19.

## Vitamin D deficiency, lupus anticoagulant‐associated thrombosis and COVID‐19

Venous and arterial thrombo‐embolic events are common in severe COVID‐19, affecting 28% of cases admitted to intensive care, despite thromboprophylaxis, in an Italian case series of 388 patients [98]. There has been considerable interest in the effects of vitamin D on coagulation but large studies have failed to show an impact of vitamin D status or supplementation on the risks for cardiovascular disease or thromboembolism [99, 100].

A much stronger case can be made though for a protective effect of vitamin D against thrombotic complications of the anti‐phospholipid syndrome [101], and here there are intriguing parallels with the thrombotic tendency in COVID‐19 [102]. Studies have shown a prevalence of up to 70% for vitamin D deficiency amongst patients with anti‐phospholipid syndrome and meta‐analysis of 4 case–control studies including 325 cases and 507 controls showed an odds ratio of 3.06 (*P* < 0.001) for frequency of vitamin D deficiency in patients with anti‐phospholipid syndrome [103]. In keeping with an effect of vitamin D, marked seasonality has been shown for anti‐phospholipid antibody titres in healthy controls with lower levels in summer months [104].

A systematic investigation of 56 patients hospitalized for COVID‐19 found 25 (45%) positive for lupus anticoagulant on the basis of coagulation tests (dilute Russell’s viper venom time (DRVVT) and activated partial thromboplastin time (aPTT) although anticardiolipin or anti‐beta2‐glycoprotein antibodies were only detected in 10% [105]. Another study reported that 20% of 216 patients positive for SARS‐CoV‐2 were found to have a prolonged activated partial thromboplastin time (aPTT) and when most of these were further tested 91% were positive on lupus anticoagulant assay [106]. It is well recognized that lupus anticoagulant activity may develop transiently, typically for two to three months, in other viral infections [107].

## Seasonal variation of vitamin D deficiency and implications for the COVID‐19 pandemic

In the absence of vitamin D supplementation, there is marked seasonal variation in vitamin D levels. In the UK, for example, sunlight does not contain sufficient UVB to allow skin vitamin D synthesis until April and in northern Europe blood levels in nonsupplemented individuals may not rise substantially until late May or June [6]. Similarly, because of its relatively long half‐life in fat stores of 2–3 months, levels in the Southern Hemisphere typically do not drop until June. Moreover, older people and people with dark skin have much lower dermal synthesis of vitamin D in response to UVB [108]. The seasonality of respiratory virus infections is of course very well documented for influenza, human coronavirus and respiratory syncytial virus (RSV) – the ‘winter viruses’ [109], although other factors such as temperature and humidity are likely also to underlie this. There is sound evidence linking vitamin D deficiency with risk for or severity of influenza [76, 110] and RSV [51, 75]. If COVID‐19 severity is strongly related to vitamin D status, this too may prove to be a winter virus, since more severe COVID‐19 illness probably results in a longer period of infectivity. Current lock‐down measures could of course blunt the normal summer rise in vitamin D.

## Vitamin D in the COVID‐19 pandemic – current knowledge

There are very limited peer‐reviewed studies currently published, and the current data are ‘soft’. The simplest but possibly the most informative is a questionnaire‐based study in Italian patients with Parkinson’s disease (*n* = 1486) and their family members (‘controls’ *n* = 1207) [111]. One hundred and five (7.1%) of patients and 92 (7.6%) family members had confirmed or probable COVID‐19. Vitamin D supplements had been taken by 13/105 (12.4%) COVID‐19 cases compared with 316/1381 (22.9%) unaffected – after age adjustment OR 0.56 (95% CI 0.32–0.99; *P* = 0.048) for vitamin D supplements reducing odds of COVID‐19 infection.

Another study from Italy has reported serum vitamin D levels taken with 7 weeks of SARS‐CoV‐2 PCR testing – mostly with 3 days of test [112]. Amongst 107 patients with available data, the 27 SARS‐CoV‐2 positives had median 25(OH)D 11.1 ng mL^−1^ (IQR 8.2–21.0) compared with the 80 SARS‐CoV‐2 negatives who had median 25(OH)D 24.6 ng mL^−1^ (IQR 8.9–30.5; *P* = 0.004). Because of the proximity of vitamin D assay to PCR testing, it is possible that vitamin D levels could have been lowered as a consequence of a negative acute phase response.

It should be noted that both of these studies are looking at the possible impact of vitamin D on risk for infection. No peer‐reviewed studies have yet been published looking at possible impacts of vitamin D on COVID‐19 severity.

Several preprints that have not yet undergone peer review are available online, but some of these are problematic. One of the more complete studies reports a retrospective cohort from Chicago of 4314 patients tested for COVID‐19 all of whom had a vitamin D level in the year before testing [113]. In multivariate analysis that adjusted for age and ethnicity, being likely vitamin D deficient (previous deficient level and lack of increased treatment) increased risk of testing positive for COVID‐19 (RR 1.77, *P* < 0.02). It should again be noted that this addresses whether vitamin D impacts on risk for infection but does not inform about risk of COVID‐19 severity.

Three studies, two currently online as preprints and one peer‐reviewed, have used historical vitamin D levels, measured between 2006 and 2010 in individuals sampled for the UK Biobank. These studies have shown no association between historical vitamin D levels (season‐adjusted) and testing positive for COVID‐19 but have not yet assessed COVID‐19 severity [114, 115, 116].

Studies are urgently needed that examine COVID‐19 outcomes in relation to vitamin D status or supplementation. An additional problem that needs addressing is possible confounding by a ‘healthy user’ effect, that is people with higher vitamin D levels possibly leading a healthier lifestyle in other ways. Care should be taken to adjust for relevant confounders such as deprivation, smoking and exercise.

## Implications for current guidance

Many countries have recommendations for use of vitamin D supplements. Current UK guidance at https://www.nhs.uk/news/food‐and‐diet/the‐new‐guidelines‐on‐vitamin‐d‐what‐you‐need‐to‐know/ is that ‘adults and children over the age of one should have 10 µg (400 International units [IU]) of vitamin D every day. This means that some people may want to consider taking a supplement’. Recommendations from other countries vary, and the World Health Organization recommends a daily intake of 5 µg (200 IU) for adults but rising to 15 µg (600 IU) over 65 years [6]. The proportion of the population taking supplements is largely unknown although in one Irish study only 4% of men and 15% of women over 50 regularly took supplements [87].

The relevant advisory bodies: European Food Standards Agency, UK Scientific Advisory Committee on Nutrition and US Institute of Medicine, all consider an upper intake limit of 4000 IU day^−1^ in adults [7]. The consensus recommendations for vitamin D intakes for older adults or those with little sun exposure (household or confined) are a daily intake of 10–20 µg (400–800 IU day^−1^). Due to an inadequate dietary intake and lack of mandatory fortification in Europe and the United Kingdom, a vitamin D supplement may be required to achieve this. However, lifestyle and demographic factors also need to be taken into account and the ‘one size fits all’ will not achieve sufficient blood 25(OH)D concentrations across all people in a similar timeframe. For instance, the response to vitamin D supplementation is blunted in those who are overweight or obese [117, 118, 119, 120]. Furthermore, men possibly have a lower response to supplementation than women [117] and people with intestinal inflammatory conditions such as Crohn’s disease have poorer absorption. Baseline vitamin D status is also critical, and older adults may have a lower response [121]. Consequently, those with very low vitamin D concentrations, obesity, chronic intestinal disease or other conditions that affect vitamin D metabolism require either a much longer run‐in time period of supplementation or a higher dose to reach a sufficient level in the same timeframe.

Identification of appropriate supplementation dose depends on the target serum 25(OH)D concentration. Although >25 or 30 nmol L^−1^ is generally accepted as adequate for musculoskeletal health, it is recommended by the US Institute of Medicine that a higher level, >50 nmol L^−1^, should be achieved [118]. Some authorities would recommend a still higher target level of >75 nmol L^−1^, but this is controversial. Extrapolation from a large number of cohort studies suggests that for adults, supplementation with 1000 IU day^−1^ should be adequate, even in obese individuals, for achieving >50 nmol L^−1^. However, to achieve >75 nmol L^−1^ would typically require supplementation of between 3000 IU day^−1^ and 4000 IU day^−1^ for an obese individual [118].

Finally, consideration also needs to be given to other dietary components that are required for vitamin D metabolism/function. For instance, the metabolism of vitamin D into the active form is a magnesium‐dependent process, whilst it also acts a co‐factor for vitamin D binding protein [122]. Dietary intakes of magnesium have been highlighted as low in both the US and UK populations [123, 124], and the recommended intakes are around 420 mg day^−1^ for men and 320 mg/day^−1^ for women [125].

## Implications for research

Research should include urgent observational studies comparing blood levels of vitamin D in the population with subsequent outcomes in COVID‐19 illness, but the caveat that vitamin D levels may fall during the acute phase response of a pyrexial illness may make interpretation difficult. Assay of free vitamin D may largely get around this problem. Simple observational studies of associations between prior vitamin D supplementation and COVID‐19 outcomes could be the fastest route to useful evidence.

Randomized controlled trials are regarded as the ‘gold standard’ for evidence but are probably harder to conduct when the intervention under test is an established vitamin rather than the more usual trial of a novel and potentially riskier drug. There are currently eleven clinical trials of vitamin D in COVID‐19 registered on clintrials.gov (Table 3). Nine of these are in symptomatic patients. Supplementing vitamin D in people who are already ill might be too late to be effective, although certainly worthy of study. Two studies will address prophylaxis, one in people >60 who are institutionalized (Lille) and the other in healthcare workers and relatives of affected patients (Tehran). Results from some of these studies will hopefully become available over the next few months.

**Table 3:** Trials of vitamin D in COVID‐19 registered on clintrials.gov (5 June 2020)

Clinical trial number	Title	Location	Subjects	Intervention	Proposed sample size	Primary outcome measure	Estimated primary completion
NCT04411446	Cholecalciferol to improve the outcomes of COVID‐19 patients (CARED)	Argentina	Nonsevere, symptomatic and hospitalized	Single oral dose of 500 000 IU oral vitD3 vs placebo	1265	Need for respiratory support and change in respiratory SOFA^a^ score	Dec 2020
NCT04407286	Vitamin D testing and treatment for COVID‐19	Arizona USA	Nonsevere symptomatic patients with low levels of vitamin D	Open‐label cholecalciferol 10 000 IU day^−1^ bd (age 18–69 years) or 15 000 IU day^−1^ tds (age 70+) for 2 weeks. Continue after 2 weeks at this dose if deficient. If insufficient after 2 weeks, 5000 IU day^−1^	100	Normalization of vitamin D levels and change in severity of COVID‐19 symptoms from baseline to 2 weeks	Aug 2020
NCT04395768	International ALLIANCE study of therapies to prevent progression of COVID‐19	Australia	Symptomatic COVID‐19	Multiple treatments including hydroxychloroquine, azithromycin, zinc, vitamin D3 5000 IU daily for 14 days, vitamin B12 with or without vitamin C	200	Change in severity and duration of symptoms, length of hospital stay and need for mechanical ventilation or mortality within 15 days	May 2021
NCT04386850^b^	Oral 25‐hydroxyvitamin D3 and COVID‐19	Tehran, Iran	Symptomatic COVID‐19	Oral 25‐hydroxy vitamin D3 25 mcg od for 2 months	1500	Hospitalization, disease duration, death and need for oxygen support	Nov 2020
NCT04386850^b^	Oral 25‐hydroxyvitamin D3 and COVID‐19	Tehran, Iran	Healthcare provider or a close patient relative with a negative COVID‐19 test living with COVID‐19‐positive patients	Oral 25‐hydroxy vitamin D3 25 mcg od for 2 months	1500	Diagnosis of COVID‐19 infection of any severity, hospitalization, disease duration, death and need for oxygen support	Nov 2020
NCT04385940	Vitamin D and COVID‐19 management	Alberta USA	Nonsevere. symptomatic patients	Daily low dose (1000 IU day^−1^) compared to weekly high dose (ergocalciferol 50 000 IU twice during first week and one dose over second and third weeks)	64	Symptom recovery (time from intervention to day 21)	Aug 2020
NCT04366908	Prevention and treatment with calcifediol of COVID‐19 coronavirus‐induced acute respiratory syndrome (SARS)	Cordoba, Spain	18–90 COVID‐19 pcr diagnosis	Best available therapy (BAT) plus calcifediol 266 µg ×2 on day 1, then ×1 on days 3,7,14,21,28 vs BAT	1008	Admission to ITU or death by day 28	July 2020
NCT04363840	The LEAD COVID‐19 trial: Low risk, early aspirin and vitamin D to reduce COVID‐19 hospitalizations	New Orleans USA	COVID‐19 diagnosis < 24 h	50 000 IU VitD3 oral Once weekly ×2 plus aspirin 81 mg od (both arms)	1080	Hospitalization within 2 weeks	Dec 2020
NCT04351490	Impact of zinc and vitamin D3 supplementation on the survival of institutionalized patients infected with COVID‐19	Lille, France	>60 institutionalized	Zinc gluconate 15 mgs ×2/day VitD 2000 IU day^−1^ for 2 months vs usual care	3140	Survival 2 months	July 2020
NCT04344041	COVID‐19 and vitamin D supplementation: a multicentre randomized trial of high‐dose versus standard‐dose vitamin D3 in high‐risk COVID‐19 patients (CoVitTrial)	Angers, France	High risk ≥ 70 year diagnosed within 48 h	Vit D 400 000 IU single dose versus Vit D 50 000 IU single dose	260	All‐cause mortality 14 day	July 2020
NCT04334005	Vitamin D on prevention and treatment of COVID‐19	Granada Spain	Nonsevere symptomatic	Single dose 25 000 IU oral vitD3 vs usual care	200	All‐cause mortality	June 2020

^a^ SOFA‐sequential organ failure assessment score.

^b^ NCT04386850 has two cohorts: a treatment study for COVID‐19‐positive patients and a prevention study for healthcare providers (HCP) or close patient relatives living with COVID‐19‐positive patients.

## Conclusions

Urgent research is needed to assess whether vitamin D deficiency is associated with increased COVID‐19 severity and to determine the effects of vitamin D supplementation. Meanwhile, given the strong circumstantial and biological evidence, and the relative safety of vitamin D supplementation, it seems sensible to advocate its use more widely during this pandemic, particularly for all those people at risk of vitamin D deficiency. The potential gain if the hypothesis is correct would be massive.

These points are summarized in Table 4.

**Table 4:** Summary

Vitamin D deficiency as a possible factor determining COVID‐19 severity Lower population mortality in countries South of 28 degrees N latitude where there will have been sufficient sunlight to maintain vitamin D levels during the past months. Vitamin D deficiency correlates with hypertension, diabetes, obesity, ethnicity and institutionalization all of which are features associated with increased risk of severe COVID‐19. Vitamin D moderates inflammatory cytokine response by macrophages and respiratory epithelial cells to pathogens including respiratory viruses. Vitamin D’s effect on cytokines and reduced risk for experimental lung injury is likely mediated by its increase in ACE2:ACE ratio and consequential reduction of angiotensin II – highly relevant to COVID‐19 since ACE2 is the SARS‐CoV‐2 receptor. Vitamin D deficiency and vitamin D receptor polymorphisms are associated with increased risk of severe viral bronchiolitis in infants. Vitamin D deficiency is easily prevented by supplementation which is very safe.

## Conflict of interest statement

JMR with the University of Liverpool and Provexis UK holds a patent for use of a soluble fibre preparation as maintenance therapy for Crohn’s disease plus a patent for its use in antibiotic‐associated diarrhoea. Patent also held with the University of Liverpool and others in relation to use of modified heparins in cancer therapy. SS has received speaker fees from MSD, Actavis, AbbVie, Dr Falk pharmaceuticals, Shire and received educational grants from MSD, AbbVie, Actavis and is an advisory board member for AbbVie, Dr Falk pharmaceutics and Vifor pharmaceuticals. EL, GG and RAK have no conflicts to declare.

## Funding

None.

## Authors and contributions

All authors contributed to conception of the article. JMR and SS wrote the first draft, and all authors contributed to revision and approved the final version.

## Author Contribution

**Jonathan Rhodes:** Conceptualization (equal); Formal analysis (lead); Writing‐original draft (lead); Writing‐review & editing (equal). **Sreedhar Subramanian:** Conceptualization (supporting); Writing‐original draft (supporting); Writing‐review & editing (equal). **Eamon Laird:** Conceptualization (supporting); Writing‐original draft (supporting); Writing‐review & editing (equal). **George Griffin:** Conceptualization (equal); Writing‐original draft (supporting); Writing‐review & editing (equal). **Rose Anne Kenny:** Conceptualization (equal); Writing‐original draft (supporting); Writing‐review & editing (equal).
